# The Role of Nanoparticles in Response of Plants to Abiotic Stress at Physiological, Biochemical, and Molecular Levels

**DOI:** 10.3390/plants12020292

**Published:** 2023-01-07

**Authors:** Jameel Mohammed Al-Khayri, Ramakrishnan Rashmi, Rutwick Surya Ulhas, Wudali N. Sudheer, Akshatha Banadka, Praveen Nagella, Mohammed Ibrahim Aldaej, Adel Abdel-Sabour Rezk, Wael Fathi Shehata, Mustafa Ibrahim Almaghasla

**Affiliations:** 1 Department of Agricultural Biotechnology, College of Agriculture and Food Sciences, King Faisal University, Al-Ahsa 31982, Saudi Arabia; 2 Department of Life Sciences, CHRIST (Deemed to be University), Bangalore 560 029, Karnataka, India; 3 Faculty of Biological Sciences, Institute of Biochemistry and Biophysics, Friedrich-Schiller-Universität, Furstengraben 1, 07743 Jena, Germany; 4 Virus & Phytoplasma Research Department, Plant Pathology Research Institute, Agricultural Research Center, Giza 3725005, Egypt; 5 Department of Arid Land Agriculture, College of Agriculture and Food Sciences, King Faisal University, Al-Ahsa 31982, Saudi Arabia; 6 Plant Pests, and Diseases Unit, College of Agriculture and Food Sciences, King Faisal University, Al-Ahsa 31982, Saudi Arabia

**Keywords:** climate changes, abiotic stress, nanoparticles, molecular changes, biochemical changes

## Abstract

In recent years, the global agricultural system has been unfavorably impacted by adverse environmental changes. These changes in the climate, in turn, have altered the abiotic conditions of plants, affecting plant growth, physiology and production. Abiotic stress in plants is one of the main obstacles to global agricultural production and food security. Therefore, there is a need for the development of novel approaches to overcome these problems and achieve sustainability. Nanotechnology has emerged as one such novel approach to improve crop production, through the utilization of nanoscale products, such as nanofertilizer, nanofungicides, nanoherbicides and nanopesticides. Their ability to cross cellular barriers makes nanoparticles suitable for their application in agriculture. Since they are easily soluble, smaller, and effective for uptake by plants, nanoparticles are widely used as a modern agricultural tool. The implementation of nanoparticles has been found to be effective in improving the qualitative and quantitative aspects of crop production under various biotic and abiotic stress conditions. This review discusses various abiotic stresses to which plants are susceptible and highlights the importance of the application of nanoparticles in combating abiotic stress, in addition to the major physiological, biochemical and molecular-induced changes that can help plants tolerate stress conditions. It also addresses the potential environmental and health impacts as a result of the extensive use of nanoparticles.

## 1. Introduction

Abiotic stress is the term used to describe how nonliving elements negatively affect living things in a particular environment. Drought, salt, heavy metals, extremely low or high temperatures and other environmental extremes are some of the potential stresses that are major global issues. In the changing global climate, plants are more susceptible to abiotic stress. Climate change, which is a result of global warming, is accompanied by a sharp increase in the frequency and severity of heat waves, droughts and other abiotic stress situations, such as flooding, salinity and freezing [1]. In addition to natural causes, anthropogenic perturbations of the biosphere, manifesting in a wide range of global phenomena, such as accelerated rate of industrialization, intensive agriculture and extensive mining, coupled with a burgeoning population and rapid urbanization, have led to catastrophes in global warming, thus indirectly contributing to abiotic stress and the grave contamination of the essential elements of life on the planet [2].

Plants are sessile organisms and abiotic stressors, including drought, salt and severe temperatures, must be endured by them [3]. Stress responses are complex, integrated circuits rather than simple, linear routes, incorporating several pathways, particularly cellular compartments, tissues and interactions with extra cofactors and/or signaling molecules to coordinate a specific response to a given stimulus [4]. The tissue or organ that is under stress will determine how the plant reacts. Additionally, the complexity of the response can be significantly influenced by the intensity and duration of the stress [5]. Plants activate early stress-signaling mechanisms in response to abiotic stresses in order to counteract stress reactions and promote tolerance [6,7]. When a plant cell detects stress, second messengers, such as calcium, reactive oxygen species (ROS), phospholipids and nitric oxide (NO), as well as various protein kinases, relay and amplify the signal [8]. SnRk1 kinases change the expression of approximately 1000 stress-responsive genes, facilitating the restoration of homeostasis by suppressing energy-intensive processes and encouraging plant stress tolerance. This enables them to resist various abiotic challenges, such as salt, flooding, drought and nutrient scarcity [9]. Plant hormones, such as ABA and ethylene, also act as primary signals for plant defense responses, such as the closing of stomata during drought stress [5]. These stress-signaling mechanisms activate transcription factors, which further activate various stress-response genes to cope with the harsh effects of abiotic stress.

The elevated ROS as a response to abiotic stress may damage proteins, membranes and other structural components of plants, which can eventually lead to impairments in their essential physiological processes [10]. Membrane peroxidation and damage to photosynthetic systems were reported in various plants, irrespective of the stress. Hence, plant defense mechanisms mainly focus on scavenging the ROS by activating antioxidant molecules [11]. Enhanced production of phenols and flavonoid is a common activity reported in many plants under various abiotic stresses. Phytochelatin production in marked amounts was observed mostly in various heavy metal stresses [12]. Proline content was also found to be increased to cope with various abiotic stresses by acting as an osmolyte. Another important biochemical change is the activation of antioxidant enzymes to scavenge the ROS molecules. SOD, APX, GPX and catalase are some of the antioxidant enzymes that help plants tolerate oxidative stress [10,12].

**Figure 1:**
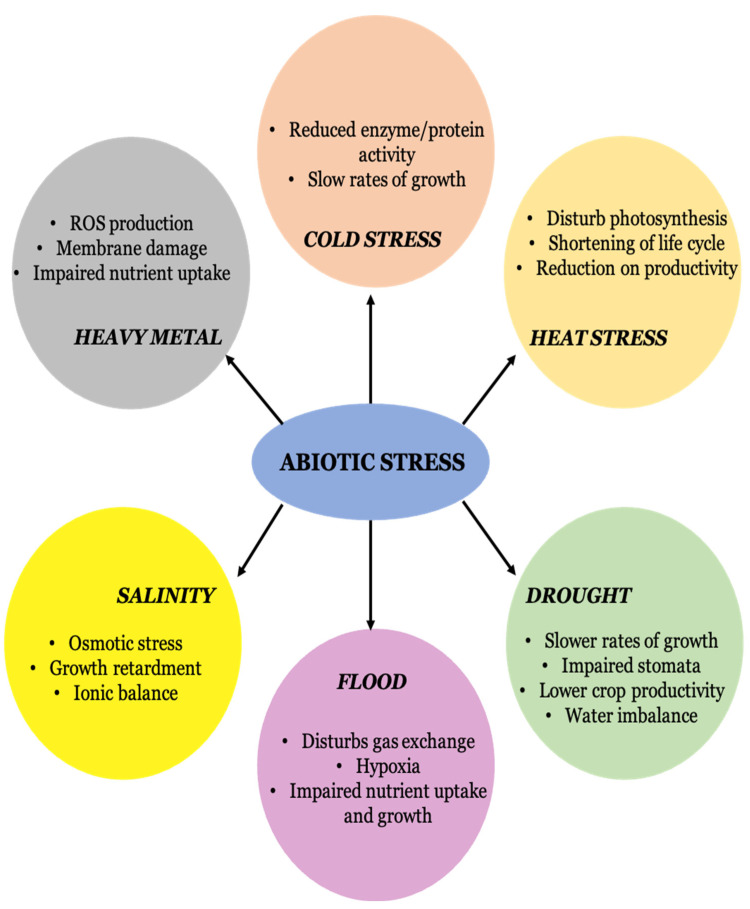
Abiotic stress factors and their effects on plants.

Long-term stressors may have negative impacts on plant development and growth, which directly affect agricultural yield. Given that plants are considered the living kingdom’s producers, conditions that have an adverse effect on plants raise concerns regarding food security [13]. Abiotic stressors have a significant role in yield losses, accounting for up to 50% of losses in the yield of major crops [14]. In this context, scientists are concentrating on research into approaches such as genetic engineering and plant breeding to reduce abiotic stress. Recently, nanotechnology also found its application in abiotic-stress tolerance [15]. Worldwide interest in nanomaterials is currently growing as a means of defending plant growth from abiotic challenges, such as drought, salt, heavy metals, extremely high temperatures and flooding [16]. Nanoparticles (NPs) are thought to be useful and promising techniques for modifying crop yield by enhancing a plant’s ability to tolerate abiotic stress, in order to address present and upcoming production restrictions in sustainable agriculture [17]. Figure 1 represents various abiotic stress factors and their responses in plants.

## 2. Absorption and Translocation of Nanofertilizers or Nanoparticles by Plants

The interaction and penetration of nanoparticles into the plant system is the preliminary step in the processes of absorption and translocation and for the mechanism of action. Osmotic pressure and capillary forces play a major role in the absorption of nanoparticles from the root epidermal regions. Usually, nanoparticles within a range of 3–5 nm are easily absorbed. With the help of small pores, nanoparticles cross the epidermal cell wall of roots and enter the plant system. In some instances, a small number of nanoparticles enhance the ability to form their own pores on the cell wall if their size is larger than that of the usual pores that absorb the nanoparticles [18,19]. In some instances, the charge of the nanoparticles plays a major role in the initial interaction that occurs with the epidermis region [20]. Furthermore, the nanoparticles take both apoplastic and symplastic pathways to reach the target tissue. Usually, the membrane carrier protein accompanies the nanoparticles and helps in transportation using the xylem channels [21]. Later, if there is any aggregation at various regions of the channels, they are sent back to the roots with the help of phloem. The cuticle and stomata are the means of passage through which the nanoparticles reach the internal system of the leaf. Particles less than 5 nm take the cuticular pathway, while particles larger than 5 nm take the latter pathway. Compared to the root, the leaf has a similar internal transport system. The nanoparticles are delivered by phloem tubes via both apoplastic and symplastic pathways to the intended location or organs such as shoots, roots and fruit [22]. 

## 3. Nanoparticles and Their Applications in Agriculture

**Figure 2:**
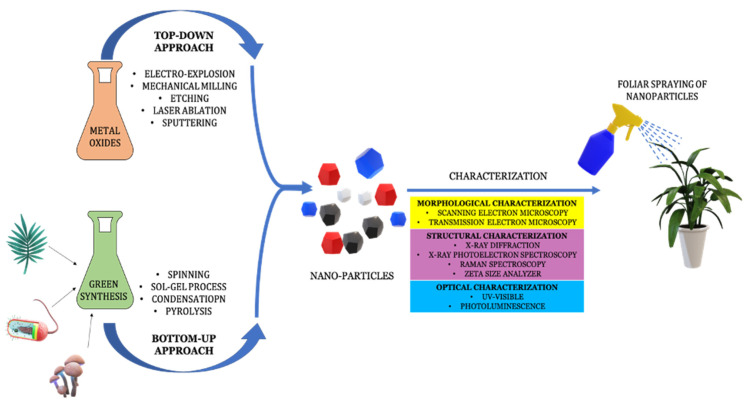
Different approaches for synthesis of nanoparticles and their characterization.

Nanoparticles have increased in their significance in a number of technological, medical, and agricultural domains. Nanotechnology’s most recent developments have aided in pushing the limits to enhance the betterment of humankind. As the name suggests, nanoparticles vary from 1 to 100 nm in size and have unique physicochemical properties [23]. Usually, nanoparticles are synthesized using top-down and bottom-up synthesis routes (Figure 2). In the top-down approach, nanoparticles are formed from larger molecules after decomposition. Electro-explosion, mechanical milling, chemical ways of etching, laser ablation and sputtering are some of the examples of top-down approaches. The synthesis of metal-oxide nanoparticles comes from top-down approaches. Whereas in bottom-up synthesis, simpler substances combine and react to form nanoparticles. Spinning, the sol-gel process, condensation and pyrolysis are some of the examples of bottom-up approaches. The green synthesis of nanoparticles, usually from biological samples, falls under the bottom-up category [24]. The following characterizations are crucial to the success of nanoparticle synthesis: 1. Morphological characterizations using microscopy (i.e., scanning electron microscopy and transmission electron microscopy). 2. Structural characterizations using XRD, XPS, Raman and Zeta-size analyzers. 3. Optical characterizations using UV-visible and photoluminescence [23]. In green synthesis, processed extracts of plants, fungus and microbes are made to react with metal salt solutions and are further processed by reduction and capping, resulting in the formation of metal nanoparticles coated with desired biomolecules. Since the size of green-synthesized nanoparticles is very small, they aid in the effective transport to the target site and also aid in improving outcomes [25]. 

As is well-known, the population of the world is growing every day, which means the way food is produced needs to be updated to keep up with demand. One method for boosting production was the use of chemical fertilizers, which eventually had a negative impact on soil fertility. Therefore, the extensive use of fertilizers results in numerous aberrations. The majority of NPK (nitrogen, phosphorus and potassium) components will, however, be depleted depending on the fertilizer content and application method owing to hydrolysis, leaching, evaporation and phytolytic degradation, eventually defeating the purpose of using them [26]. On the other hand, the use of nanoparticles in agriculture has gained importance in recent times. Nanoparticles were treated as ecofriendly and nontoxic and had a large impact on the reduction of pathogens in agriculture. Nanotechnology has also helped in the design of nanofertilizers and pesticides, which were green synthesized and coated with various traditional chemical compounds that were generally used as pesticides and fertilizers [27]; furthermore, nutrients can be encapsulated inside nanotubes or materials [28]. Nanofertilizers or nanoparticles have increased surface area and contact ratios, increasing bioavailability and helping in the easy dispersion of the desired micro or macro nutrients, which ultimately increases the uptake efficiency by the target tissues. Nutrients, soluble fertilizers coated with semipermeable membranes and waxes help in the controlled release of nutrients at the target site, which also helps to improve ecological balance [29,30]. 

Numerous studies back up the use of nanoparticles/nanofertilizers, which, when used in both in vitro and in vivo approaches, eventually aid the plant’s overall growth and development. Treatment of ZnO nanoparticles at 1000 ppm concentration helps with seed germination, shoot and root development in peanut plants [31]. When 1.5 ppm ZnO nanoparticles were sprayed over chickpea leaves, it was found that seed-germination ability rose and biomass accumulation improved [32]. Multiwalled carbon nanotubes showed better efficiency in the growth of tobacco cells [33]. The synergistic effect of SiO_2_ and TiO_2_ nanoparticles increased the uptake capacity of water and fertilizers [34]. SiO_2_ nanoparticles, when treated with *Zea mays* L., showed increased efficiency in the production of biochemicals and better photosynthetic efficiency and protein production [35]. In addition, TiO_2_ nanoparticles also helped to increase photosynthetic efficiency and in the better accumulation of biomass in spinach [36]. Similarly, TiO_2_, when treated with seeds of *Brassica napus*, was observed to increase seed-germination efficiency [37]. Apatite nanoparticles (hydroxyapatite) were synthesized using a chemical method. When treated with soybean cultures, the seed yield and growth ratio were increased drastically when compared with conventional Ca(H_2_PO_4_)_2_, phosphorus fertilizer [38]. Other than as nutrients and fertilizers, plant growth regulators are also used or encapsulated in nanomaterials to increase the potential in agriculture. Polymeric nanoparticles were prepared using gibberellic acid formulated along with chitosan and alginate. It was observed that these polymeric nanoparticles lead to an almost four-fold increase in fruit production [39]. 

## 4. Nanoparticles in Genetic Engineering of Plants

The development of diverse gene-transformation technologies has been assisted by contemporary biotechnology. Nanotechnology has also showed promise in the development of a novel method of gene transformation employing nanoparticles, along with the biolistic gun/gene gun and Agrobacterium-mediated techniques. The chromosomal DNA of *Zea mays* was incorporated into Cre recombinase and loxP sites with the help of mesoporous silica nanoparticles. The transformation was successfully established, and the desired editing at the gene level was achieved [40]. The layered double hydroxide (LDH) clay nanoparticles might be loaded with the biodegradable dsRNA of different plant viruses. dsRNA provides protection against the cauliflower mosaic virus (CMV) in treated tobacco leaves, and this protection was additionally retained in newly developed leaves [41]. In pollen grains of cotton, the β-glucuronidase-gene-coated magnetic nanoparticle complex was used for gene transformation and it was clearly observed that offspring stably inherited the characteristics of the incorporated genetic material [42].

## 5. Nanoparticles in Abiotic Stress Management

### 5.1. Nanoparticles in Salt-Stress Tolerance

**Figure 3:**
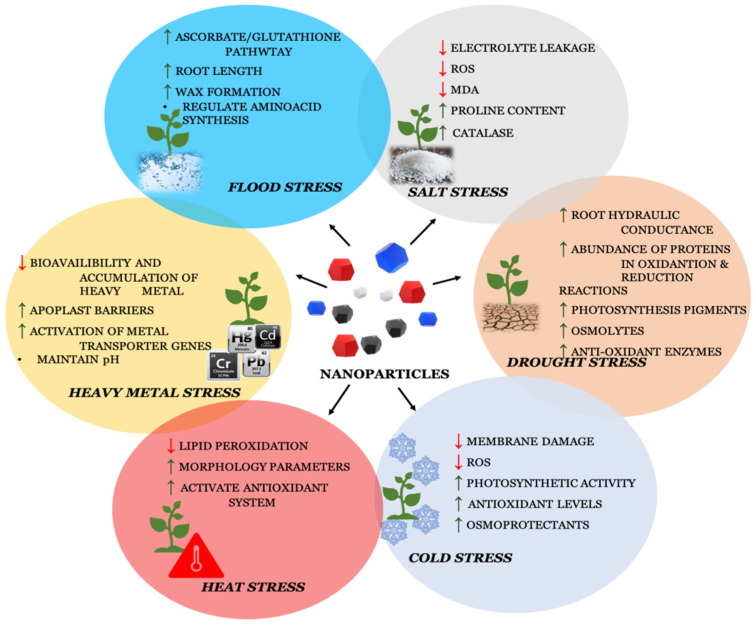
Nanoparticles involved in combating abiotic stress.

Global-warming-driven water scarcity also forces irrigation with saline water in agricultural lands all over the world, which leads to enhanced salt content in the soil. Salinity (the buildup of excessive salt in the soil) is one of the main challenges to modern agriculture, and it eventually stunts and impairs plant growth and development, ending in plant mortality [43,44]. Most plants die when the NaCl content is higher than 200 mM. Salinity has a significant impact on every stage of the plant’s life cycle, including seed germination, seedling development, vegetative growth and blooming [45]. Numerous horticultural crops, such as fruits, vegetables and spices, are impacted by salinity. In addition to causing osmotic stress, water stress, oxidative stress, nutritional stress and reduced cell division, salt stress imbalances ionic strength, which has an impact on a number of biochemical, physiological and metabolic processes [46,47]. The response to various abiotic stress has been illustrated in Figure 3.

According to Zulfiqar and Ashraf [48], the application of nanoparticles, such as Zn NPs, Ag NPs, SiO_2_ NPs, Cu NPs, Fe NPs, Mn NPs, C NPs, Ti NPs, Ce NPs and K NPs, was effective in mitigating the toxic effects of salt stress in various plants. El-Sharkawy et al. [49] found that the foliar application of K NPs in salt-sensitive *Medicago sativa* improved salt tolerance by reducing electrolyte leakage and enhancing the proline and antioxidant-enzyme content, such as that of catalase. Similarly, reduced oxidative stress was evident in the lower MDA and ROS levels and higher antioxidant activity in AgNPs-treated pearl millet plants, which may have been caused by a decrease in Na^+^ absorption in the leaves [50]. Cerium-oxide nanoparticles were discovered to be beneficial in increasing photosynthetic activity in *Brassica napus* by altering the root cells and thus improving the mineral uptake [50,51]. Increasingly prevalent data suggest that applying nanoparticles to plants can considerably reduce the detrimental impacts of salt stress, and thus also control plant adaptations.

### 5.2. Nanoparticles in Drought-Stress Tolerance

Drought is regarded as the most detrimental environmental stress, reducing crop yield more than any other. According to the Intergovernmental Panel on Climate Change (IPCC), the average temperature will rise by 1.8 to 4.0 °C by 2100, and drought will affect vast areas of the world [52]. Drought affects agriculture when plants have insufficient moisture to develop normally and complete their life cycles. The severity of drought is further increased by a continuous decline in precipitation and increase in evapotranspiration demand [53]. For instance, drought stress prevents plant development, because water is required for cell turgor, which is the pressure that a contained liquid exerts on cell walls, causing cells to expand [8]. The principal effects of drought on crop plants include slower rates of cell division and growth, smaller leaves, longer stems and roots, disordered stomatal oscillations, altered water and nutrient relationships with lower crop output and inefficient water usage [53].

As per previous studies, NPs cause a variety of morphological, physiological and biochemical changes in plants as they increase their resistance to drought stress by increasing plant root hydraulic conductance and water uptake and demonstrate a differential abundance of proteins involved in oxidation-reduction, ROS detoxification, stress signaling and hormone pathways [17]. The foliar application of metal-oxide nanoparticles, such as titanium dioxide (TiO_2_), zinc oxide (ZnO) and iron oxide (Fe_3_O_4_), were found to be effective in enhancing the plant’s physiological and metabolic activities under drought stress [54]. When Si NPs were applied to drought-stressed pomegranate plants, additional improvements were made to their photosynthetic pigments, nutrient status, physical and chemical parameters (especially those related to fruit cracking), phenolic content and concentrations of osmolytes, antioxidant enzymes and abscisic acid [55]. El-Zohri et al. [56] suggested that green ZnO-NPs administered topically at lower concentrations could successfully boost tomato tolerance to drought stress. In addition to nanofertilizers, green synthesized Fe_3_O_4_ NPs were also found to be effective in reducing the impact of drought stress on fenugreek plants [57]. However, a study by Potter et al. [58] indicates that the potential benefits of using NPs in enhancing plant drought resistance only actualize under specific environmental circumstances.

### 5.3. Nanoparticles in Cold-Stress Tolerance

Global climate change also contributes to cold or low-temperature stress, which harms plant growth and development. Plants often experience two types of low-temperature stress: chilling and freezing. Chilling temperatures for plants range from 0 to 15 °C, depending on the species and tolerance level of the plant. The air temperature and wind speed during exposure are other factors that affect chilling temperatures. In contrast to its response to chilling temperatures, the plant will battle against freezing temperatures (below 0 °C) [59]. Crop species can be hurt or killed by low and nonfreezing temperatures, which can have an impact on their productivity, survival and ecological dispersion [60]. As enzyme and other-protein activity are reduced at colder temperatures, cold stress slows down plant growth [8]. Numerous processes in these plants are impacted by low temperatures, including those involved in secondary metabolism, respiration, defense and protein and nucleic acid production [59].

Chitosan nanoparticles and TiO_2_ NPs have been used extensively in a variety of studies for their efficiency in cold-stress tolerance. The application of Ti NPs was found to be effective in improving electrolyte leakage, photosynthetic activity and membrane damage under cold-stress conditions in chickpea plants using transcriptional regulation [61,62,63]. Hasanpour et al. [64] suggest that when TiO_2_ NPs are applied to plants, the tolerance of chickpea plants to cold stress may develop by controlling the pressure of the temperature drop injury and altered metabolism for plant growth. The deleterious effects of cold stress are reduced and glycyrrhizin content is enhanced when using TiO_2_ NPs in licorice plants [65]. The use of chitosan nanoparticles was found to be effective in reducing the ROS with the accumulation of osmoprotectants in banana plants under cold-stress conditions [66]. Furthermore, in rice plants, the foliar application of ZnO NPs may reduce chilling stress through the antioxidative system and transcription factors involved in the chilling response [67]. Similarly, the use of SiNPs can also improve the photosynthetic ability of sugarcane plants under chilling stress [68].

### 5.4. Nanoparticles in Heavy-Metal-Stress Tolerance

Heavy-metal (HM) stress is one of the deleterious factors that reduces crop productivity in the modern day. Human activities, such as industrialization and urbanization, have resulted in HM pollution all over the world [2]. Enhanced implementation of modern agricultural tools, such as chemical pesticides and fertilizers, has also contributed to HM stress in crop plants. Heavy metals such as Hg, Pb, Cd, Ni, Co, Cr and Ag have deleterious impacts on plants [69]. Since plants reside at the baseline of trophic systems, the chances of bioaccumulation of these HMs via the food chain are high, and this eventually leads to chronic health impairments, such as kidney and liver damage, in humans and other animals. In addition, HMs have a direct impact on plants, such as through morphological and physiological abnormalities and impaired metabolic pathways [70]. These affect the quality and quantity of plant-based products, especially in agricultural crops and medicinal plants.

A number of studies on the use of nanoparticles to alleviate HM stress have been conducted. Nanoparticles applied to the soil can absorb and transform the HMs in soil, thereby reducing the bioaccumulation and mobility of HMs. The Cd metal availability in soil has been reduced by the application of Fe_3_O_4_ NPs [71]. The hydroxyapatite NPs can reduce the toxic effects of metals in soil and can maintain the soil pH by releasing phosphate ions [72]. NPs also induce the formation of apoplast barriers, which reduce the heavy-metal content in the root. Furthermore, heavy metals can be intercepted by the regulation of metal transporter genes in plants using specific NPs, which can deter the translocation of HMs by forming complexes with them [73]. NPs such as SiNPs have endorsed the production of organic acids that curtail the damage of HM stress [74,75]. NPs also activate the antioxidant system, thereby reducing the stress caused by ROS [75].

### 5.5. Nanoparticles in Flooding-Stress Tolerance

Most plants are sensitive to flooding as a result of excessive water clogging in soil. Flooding is caused either by excessive rainfall, poor soil drainage or irrigation practices. The complete submersion of plants in floodwater can be disastrous for crops. Flooding is thus one of many abiotic-stress factors that affect food availability and countries’ economies. It influences the plants grown in different ecosystems, such as floodplains, riparian zones, salt marshes, tidal zones and wetlands. Plants grown in different ecosystems show varied responses to flooding stress; wetland plant species show tolerance to shoot submergence and soil water logging, while dry-land species are sensitive to flooding stress. Excessive water logging in air spaces delays the exchange and diffusion of gas between the roots (rhizosphere) and the atmosphere, thereby inhibiting respiration due to a lack of oxygen leading to hypoxia and ultimately leading to anoxia in plants. Under flooding stress, soil pH and redox potential will be affected, the carbon-dioxide content increases and the mobilization of phytotoxins increases, affecting the root metabolism, nutrient uptake and overall plant growth [76].

Nanoparticles have been reported to alleviate flooding stress in plants. In soybean plants under flooding stress, silver NPs helped to alleviate stress conditions by regulating amino-acid synthesis, proteins, glycolysis and wax formation, and NPs enhanced the growth of soybean plants despite stress [77,78]. Another study has been conducted into soybean plants under flooding stress, where Al_2_O_3_ NPs were applied to ameliorate the growth impairment induced by flood stress. The Al_2_O_3_ NPs increased root length, including that of the hypocotyl, suppressed the proteins involved in glycolysis, arbitrated the cells involved in the scavenging of ROS by upregulating the ascorbate/glutathione pathway (AsA/GSH) and increased the ribosomal proteins [79].

### 5.6. Nanoparticles in Heat-Stress Tolerance

High temperatures can cause heat stress. In recent decades, global warming has worsened this trend. The rise in temperature above a critical limit for a longer time sufficient to permanently harm plant development is often understood to constitute heat stress [80]. Extreme changes may damage the intermolecular connections required for optimal growth during hot summers, which would hinder plant development and fruit set [81]. In general, heat stress decreases the effectiveness of photosynthetic processes, shortening the plant life cycle and lowering productivity [82]. Heat stress may become a significant issue restricting field-crop productivity in tropical and subtropical areas.

The application of Se NPs in sorghum plants exposed to high temperatures was found to be helpful in ameliorating negative impacts, such as membrane damage and reduced pollen germination and crop yields, by activating the antioxidant defense system [83]. The application of AgNPs shielded wheat plants from heat stress by enhancing morphological growth [84]. Similar to this, Zn nanoparticles were discovered to be helpful in improving wheat’s ability to withstand heat stress by increasing the production of antioxidant enzymes and decreasing lipid peroxidation [85]. The foliar application of nanoparticles on tomato leaves becomes activated when the temperature exceeds certain limits and protects plants from heat stress. Si NPs are also said to be helpful in coping with heat stress [86].

## 6. Response of Plants to Nanoparticles under Abiotic Stress

Some of the most extensively researched useful NPs include carbon nanotubes (CNTS), MWCNTS, fullerols, metal-based NPs (Ag NPs and Au NPs), crystalline powders of nanoscale (Fe, Co and Cu) and metal-oxide NPs (iron oxide, TiO_2_ NPs, ZnO NPs, SiO_2_ NPs, CuO NPs and CaCO_3_ NPs) [87]. NPs have high surface energy and a high surface/volume ratio, which improves their reactivity and increases biochemical activity that has diverse impacts on plants [88]. NPs can quickly interact with plants and stimulate molecular mechanisms [13]. In addition, the NMs function in a dual manner, first protecting against ROS while simultaneously acting as inducers of oxidative stress, which in turn causes the activation of the antioxidant defense system in plants [14]. NPs enable plants to adjust to stressful conditions more effectively and produce more yields by reducing the toxic effects of abiotic stress and by influencing various morphological, anatomical, physiological, biochemical and molecular attributes of plants.

### 6.1. Morphological Changes under the Influence of Nanoparticles

Abiotic stresses such as salt, drought, high and low temperatures and heavy metals significantly affect aspects of plant morphology, such as fresh and dry weight, leaf area, shoot and root length, overall plant growth and crop yield [84]. According to Hassanisaadi et al. [89], the priming of seeds with low concentrations of NPs increased root length, shoot length and seed germination rate. The reduced pollen germination, seed set and yield in sorghum under the influence of temperature stress were recovered by the application of Se NPs [83]. Silver NPs derived from plants contribute to the increase in leaf area and dry weight of aerial structures under high temperatures [84]. TiNPs have been reported to increase plant height in *Dracocephalum moldavica* under salinity stress [90]. FeNPs have been shown to increase plant growth and overall biomass production in *Brassica napus* under drought stress [91]. The morphological changes under the influence of nanoparticles are reported in Table 1.

### 6.2. Anatomical Changes under the Influence of Nanoparticles

Plants undergo various anatomical changes in response to abiotic stress. The anatomical responses depend on the type of abiotic stress. Under drought conditions, the structure of stomata undergoes changes (large sub-stomatal cavities) and there is an increase in the thickness of the upper-epidermal waxy cuticle, cuticular edges and xylem tracheids lignification [98]. Under heat stress, decrease in cell size, closure of stomata and transcription rate is curtailed, while an increase in stomatal and trichomatous densities with bigger xylem vessels in roots and shoots is observed [99]. The mesophyll cells have been damaged and the plasma membrane permeability has been increased in *Vitis vinifera* L. CV. Jingx under heat stress [100]. Transpirational water loss has been reduced by engaging in bimodal stomatal behavior in *Zygophyllum qatarense* Hadidi exposed to high temperatures [101]. The application of nanoparticles helps the plant survive under stress conditions by aiding in the anatomical adaptations of the plant. The TiO_2_ nanoparticles have been successful in regulating the stomatal opening in maize plants under heat stress, thus reducing its impact [102]. The treatment of SiO_2_ nanoparticles inhibited the negative effects of salinity by improving the epicuticular wax layer (EWL) in strawberry plants [103]. In the study conducted by Mustafa et al. [77], the Al_2_O_3_ nanoparticles reduced cell death in the hypocotyl region of the soybean plant. ZnO NPs were shown to be successful in reducing damages to epidermis and vascular tissues in *Sorghum bicolor* under salt stress [92]. Another study on the influence of CeO_2_ NPs in *Brassica napus* L. under salt stress has been reported. It was observed that the former could shorten root apoplastic barriers [51].

### 6.3. Physiological Changes under the Influence of Nanoparticles

Plants under stress exhibit a variety of physiological responses, such as changes in the photosynthetic machinery, transpiration, mineral and water uptake, lipid peroxidation and seed germination. Under cold stress, plants exhibit inhibition in the germination of seeds and decreased pollen fertility, seed set and chlorophyll content, thereby affecting photosynthesis [104]. Further, plant cells undergo electrolyte leakage, protoplasmic streaming and plasmolysis [105]. Heat stress can decrease the rate of nutrient uptake in plants [106]. The Cd accumulated in soil also has an effect on macronutrient and micronutrient uptake due to the inability of plants to absorb water under Cd stress [107]. Drought stress is known to inhibit photosynthesis by affecting the thylakoid membranes. High soil salinity causes the inhibition of plant germination [105]. Heavy metals also decrease seed germination and photosynthesis and have other deterring effects on various plant physiological processes [108]. Si nanoparticles increase the rate of photosynthesis and stomatal conductance in plants experiencing drought stress and help them to survive in stress conditions [109]. In tomato leaves under heat stress, the application of a fixed concentration of nano-TiO_2_ increased leaf transpiration, net photosynthetic rate and stomatal conductance. Nano-TiO_2_ decreased the energy loss from the photosystem II (PS II) and increased unregulated energy loss [102]. The physiological changes under the influence of nanoparticles are reported in Table 2.

### 6.4. Major Biochemical Changes to Tolerate Abiotic Stress

Abiotic stress such as salinity, drought, and high metal level stresses have overlapping effects on plants which includes elevated levels of reactive oxygen species, activation of the antioxidant system, and buildup of inert solutes (osmolytes), such as sugars, polyamines, secondary metabolites, and amino acids [115]. Under abiotic stress, the proteins and enzymes are susceptible to denaturation resulting in enzyme activation, the protein production reduces, the fluidity of membrane lipids increases, the membrane integrity is lost [116]. The total carbohydrate content varies in response to different biotic stresses. The total carbohydrate content is known to decrease under salinity stress while it is known to increase in response to lower temperature stress [117]. The plants under abiotic stress produce enzymes, and secondary metabolites such as anthocyanins, flavonoids, lignins, phenolic acids and other molecules to alleviate the abiotic stress and manage the oxidative stress in order to reduce cellular damage [116].

According to reports, NPs cause a variety of morphological, physiological, and biochemical changes in plants to increase their resistance to drought stress [17]. By reducing the misfolding of proteins brought on by flooding stress, the application of Ag NPs improved the ability of soybean seedlings to withstand stress [77]. The foliar application of Zn NPs boosted the levels of proline, glycine betaine, free amino acids, and sugars under drought conditions [118]. Under various abiotic stresses, proline concentration is found to be high. Proline has been hypothesized to protect enzymes and cellular structures when present in high concentrations as an osmoticum [119]. The accumulation of such osmolytes will help to maintain the redox potential of cells under stressful conditions [52] and also acts as a signal for antioxidant response [115]. The biochemical changes under the influence of nanoparticles have been reported in Table 3. 

### 6.5. Antioxidant Response 

Reactive oxygen species (ROS) are the byproducts of oxygen metabolism and oxidative stress is characterized by an increase in ROS concentration in the cell. In an attempt to mitigate the oxidative stress, the cell employs several mechanisms [120,121]. The signaling, expression profiles, metabolic model, biochemistry and physiology that a cell display during an oxidative stress, in order to equilibrate and production and detoxification of ROS and re-establish redox homoeostasis constitutes its anti-oxidant response [122]. Such an anti-oxidant response generally utilizes enzymes such as Superoxide dismutase (SOD), glutathione peroxidase (GPX), Catalase (CAT), Peroxiredoxins (Prxs) and Guaiacol peroxidase (POX). In addition, the ascorbate-glutathione cycle and its constituent enzymes are active. Along with the enzymes, ascorbate and glutathione buffers, and secondary metabolites such as carotenoids, tocopherols and phenolics are some of the non-enzymatic components that play a role in antioxidant response [123]. Oxidative stress may be indirectly acquired due to other abiotic stresses and thus this response system and its members must possess high tolerance to a wide variety of stresses. For instance, H_2_O_2_ concentration raises in a variety of stresses depending on the intensity and duration of stress and its differential abundance in various cellular compartments is disrupted depending on the type of stress [124]. In response to this, an enzyme system has evolved consisting of 152 genes in Arabidopsis which establishes a complex system-wide physiology supporting a variety of mechanisms to neutralize the stress [125]. Similar systems have also been studied in other plants including rice and wheat cultivars using multi-omics approach as well as simpler measurements of ROS and H_2_O_2_ [126,127,128]. The damage itself is generally measured based on electrolyte leakage, lipid peroxidation and propidium iodide fluorescence assay. NPs are known to both induce and complement this antioxidant response system due to its ROS generation and scavenging properties as well as its ability to regulate membrane damage. 

CeO_2_ NPs were seen to scavenge and hence reduce ROS and H_2_O_2_ concentrations in germinating rice seedlings in a dose dependent manner [129,130]. CeO_2_ NP’s surface lattice has vacant oxygen sites which allows them to scavenge O_2_- radicals and OH radicals and thus alternate their oxygen states between Ce^3+^ and Ce^4+^ [131]. At low concentrations CeO_2_ NPs suppressed ROS production in *M. arborea* while higher concentrations mimicked SOD activity [132]. Similar response was seen when *A. thaliana* was treated with CeO_2_ NPs, but in addition a 2.5-fold increase in lipid peroxidation was recorded [133].

TiO_2_ NPs in its rutile and anatase form were pretreated to spinach seeds before being planted and later sprayed with TiO_2_ NPs during oxidative stress due to UV-B irradiation and the damage were assessed in the extracted chloroplast. O_2_- radical and H_2_O_2_ accumulation and thus lipid peroxidation was found to be significantly reduced [134]. Similar to CeO_2_ NPs, the TiO_2_ NPs can alternate between its 2 oxidation states Ti^4+^ and Ti^3+^ while oxidizing or reducing the ROS and H_2_O_2_. It can also increase the tolerance in stressed chlorophyll of chickpea cultivars by reducing the electrolyte leakage [61]. In addition, TiO_2_ NPs also have the ability to generate ROS in dark and light environments, independent of its size [135]. This causes it to increase lipid peroxidation of membrane damage. For instance, in *Lemna minor* treated with TiO_2_-A NPs without oxidative stress led to membrane damage, however lipid peroxidation was not observed probably due to absorption of TiO_2_-A at the roots, which is biologically inert [136]. 

ZnO NPs have a distinct surface morphology that exposes the polar faces outwards which can then fill it’s O_2_ vacancies with electrons to produce free radicals and thus generate ROS [137]. When *A. cepa* and *Fagopyrum esculentum* was treated with ZnO NPs, it led to oxidative stress due to the NPs itself as well as the ions they released [138]. In contrast, they dissolved free Zn ions and alleviated stress in *Pseudokirchneriella subcapitata* [139,140]. NiO NPs also led to elevated lipid peroxidation and ROS generation in tomato roots [141]. Thus, could be attributed to the ability of Ni to take part in the Haber-Weiss cycle and thus generate OH radicals. However, it cannot be concluded whether these released Ni ions or the NiO NPs as a whole contributed to the ROS generation. Such an ambiguity also arises in case of ZnO NPs, CoO NPs, CoO_2_ NPs and CuO NPs as well, in terms of weather the metal ion released which can alternate between 2 oxidation states or the NPs as a whole causes ROS generation and lipid peroxidation [138,139,140,142]. CuO NPs however can form a unique pentavalent ion that can generate ROS in *Elodea densa* by the Fenton reaction [143]. It also caused loss of membrane integrity due to K^+^ leakage in hydroponic treatment in maize [144]. Fe_2_O_4_ NPs can increase lipid peroxidation by blocking aquaporins and disrupting the respiration rates in ryegrass and pumpkin roots [145]. 

Other than metal oxide NPs, Ag NPs, graphene NPs and MWCNTs also have been studied in terms of antioxidant response. Ag NPs when treated onto *B. juncea* led to large reduction in lipid peroxidation and H_2_O_2_ concentrations due to its ability to potentially increase redox reaction efficiency by acting as an electron relay center in these reactions [146,147]. In contrast, graphene NPs and MWCNTs increased H_2_O_2_ and ROS content and thus loss of cell viability when treated to cabbage, tomato, red spinach (for graphene NPs) and rice suspension cells (for MWCNTs) respectively [148,149]. 

### 6.6. Molecular and Signaling Response

When subjected to abiotic stress, a plant gives a complex manifold response whose early phase mainly involves stimulation of stress phytohormones, ROS accumulation as well as kinase cascades and activation of ion channels that communicate both functional electro-physiology as well as oscillatory signaling [150,151,152]. Following the early phase, the next system of response involves species and stress dependent complex regulation of gene networks whose downstream targets generally involve defense and repair of the stress-induced injury [153]. Some of the stress induced proteins also play a role in neutralization of ROS, induction of mitogen-activated protein (MAP) kinase and salt overly sensitive (SOS) kinase signaling cascades [123,150]. Recently, functional outcomes of these regulatory networks were also found to include ion and water transport and uptake as well as proteins that further regulate the transcription of secondary response elements [154,155]. The treatment of NPs to plants under abiotic stress is reported to alleviate the stress by inducing one or more of the molecular responses described above. Some of the well-studied NPs are Aluminum oxide (Al_2_O_3_) NPs, Silicon NPs, Zinc oxide (ZnO) NPs, multi-walled carbon nanotubes and Titanium dioxide (TiO_2_) NPs. The study conducted by Zhao et al. (2022) has concluded that nanomaterials such as silver and copper oxide have been potential nanomaterials by inducing stress responses and defense mechanisms thereby increasing crop stress resilience [156]. The molecular responses under the influence of nanoparticles have been reported in Table 4. 

#### 6.6.1. Salt Stress

Salt ion concentrations can cause toxicity to plants due to osmotic as well as oxidative stress in the cells. The osmotic dis-regulation also leads to nutrient deficiency in the plants [157]. Both Ag and Si NPs upon treatment to *Solanum lycopersicum* L. under osmotic stress, the differential transcriptome showed an upregulation of the genes for proteins such as ABA Response Element Binding Protein (AREB), Cystein-Rich Receptor-Like Protein Kinase 42-Like (CRK1), 9-Cis-Epioxycarotinoid Dioxygenase 3 (NCED3) and downregulation of the genes for proteins such as ABA and Environmental Stress-Induced Protein (TASI4), Zinc Finger Homeodomain Transcription Factor (ZFHD1), Dwarf And Delayed Flowing 2 (DDF2) and variants of MAPK. These differentially regulated genes are all involved in ABA pathways and the perturbed ABA would then lead to regulation of water and salt balance in plants by modulating stomatal closure by activating Sucrose non-fermenting 1-related protein kinase (SnRK2) [158,159].

One of the downstream targets of SnRK2 is to phosphorylate the AREB transcription factor which in turn transcribes key genes of the ABA pathway thus forming an indirect positive feedback loop [160]. SnRKs also have RBOH1 as a common phosphorylation target which also mediates ABA signaling. However, RBOH1 is also an NADPH oxidase that activates the ROS signaling network [158,161]. Thus, the NP mediated SnRKs activate ABA signaling via AREB instead of RBOH when in osmotic stress. On the other hand, in drought, the Si NPs were found to inhibit ABA signaling that activated *APX2* expression which have initiated a chloroplast electron transport chain to give rise to ROS and H_2_O_2_ [162]. AREB1, a member of the AREB transcription factor family, also plays a role in plant growth arrangement and seed germination during osmotic stress [163]. AREB1 upregulates the expression of *P5CS1* which is the key enzyme of proline biosynthesis and thus influences osmoregulation [164,165]. Proline is also an antioxidant signaling molecule that can chelate metal ions [166]. However, NPs have also been observed to directly upregulate *P5CS1* [158].

The ABA pathway is also upregulated by NCED3 during osmotic stress and TAS14 during drought stress [167]. While the ABA-dependent stress response proteins such as TAS14 and NCED3 are clearly upregulated, the gene *ZFHD1*, which was downregulated by the Ag NPs, infact codes for an ABA-independent stress response protein [168]. This indicates that NP treatment to a plant under osmotic stress selectively upregulates the ABA-dependent response systems. NPs also selectively activated ABA over other potential stress related phytohormone regulation. For instance, NPs generally downregulated DDF2 which is known to alleviate osmotic stress in plants by reducing gibberellic acid biosynthesis [169]. One of the key consequences of ABA signaling is oxidative stress alleviation [170]. Another gene which is upregulated by the NPs is CRK1, which is known to sense ROS and regulate the redox equilibrium via extracellular protein domains [171].

**Table 4:** Molecular responses under the influence of nanoparticles.

Stress	Plant Studied	Nano Particle Used	Plant Response	Reference
Salinity and osmotic stress	*Lycopersicon esculentum* Mill.	Ag, Si	ABA upregulation and its dependent stress alleviation by NCED3, TAS14 and SnRK2 upregulation; osmoregulation and oxidative stress alleviation by AREB1-mediated *P5CS1* overexpression and proline biosynthesis; SOS, ROS and MAPK-signaling activation	[158]
Drought stress	*Arabidopsis thaliana* L.	TiO_2_, Ag, MWCNTs	Noncoding RNA-mediated translational repression of physiological target genes	[172]
Flood stress	*Glycine max* (L.) Merr	Ag, Al_2_O_3_	Protein, lipid and energy metabolism perturbation by PAB2 and BKR1 upregulation and PDC downregulation, respectively, and antioxidant defense by glyoxalase II 3 downregulation	[77]
Cold stress	*Cicer arietinum* L.	TiO_2_	Upregulation of RUBISCO and PEPC to increase photosynthesis and reduce electrolyte leakage index	[63]

The major signaling pathways that are activated by NPs in osmotic stress include SOS, MAPK and ROS signaling. SOS pathway regulates salt tolerance and ion homeostasis while MAPK and ROS pathways regulate oxidative stress conditions [122]. These pathways are either activated or repressed depending on the nature of the NPs. For instance, Ag NPs activated MAPK2 while Si NPs repressed it under the same salt conditions [158].

#### 6.6.2. Drought Stress

Plants have devised two major changes to tolerate drought stress, reduction of transpirational loss of water by stomatal closure and increased root water uptake by morphological and anatomical modifications. However, very few molecular studies exit for this phenomenon and only one study involved NPs treatment. Garcia-Sanchez et al., 2015 observed 16 genes that were responsive to drought out of the 351 that were differentially regulated when A. thaliana was treated withTiO_2_ NPs, Ag NPs and multi-walled carbon nanotubes [172]. However, it can be assumed to apart from the small number of coding genes that is responsive, there could certainly be non-coding elements such as miRNAs that could regulate essential physiological genes by transcriptional repression, which is common in plants under stress [173,174]. 

#### 6.6.3. Flood Stress

Flooding greatly reduces the availability of oxygen in the submerged roots of the plant, causing drastic physiological changes and damage [175]. When *Glycine max* L. (soybeans) were exposed to Ag NPs and Al_2_O_3_ NPs during flooding stress, the differential proteome between the two NPs treated and untreated showed 172 proteins with altered concentrations of which 107 were root proteins [77]. The differential transcriptome on the other hand showed NPs induced downregulation of Flavodoxin-Like Quinone Reductase (FQR1), Alcohol dehydrogenase 1 (ADH) and Pyruvate Decarboxylase 2 (PDC) while upregulating NmrA-Like which is a negative transcriptional regulator. The alleviation of the flood stress was better with Al_2_O_3_ NPs treatment compared to Al NPs treatment [77]. 

The proteins related to energy metabolism were downregulated by the NPs and those of glycolysis, protein translation and post translational modification as well as lipid metabolism was upregulated by the NPs during flood [77]. Most importantly, glycolysis regulation is based on the O_2_ availability and the submersion induced anaerobicity that redirect the carbon flux towards fermentation [176]. The anaerobic stress due to the formation of methylglyoxal is detoxified by glyoxalase into lactate. In response to Ag NPs, glyoxalase II is downregulated along with other fermentation related proteins in the root cells indicating a possibly lowered cytotoxicity in the NP treated plants [77,177]. The conversion of acetaldehyde to ethanol is the key step in fermentation which is catalyzed by PDC exclusively during anaerobic stress in the plant cells. The lower PDC expression in NPs treated plants under flood stress compared to the untreated plants indicate that NPs have a potential role in alleviating the anaerobic stress [178,179]. Lipid metabolism is also perturbed by NPs during floods. Beta Ketoacyl reductase 1 (BKR1) catalyzes fatty acid and wax biosynthesis which is then deposited on the cuticle as a physical barrier to water stress [180]. During flood, the BKR1 expression is increased by the NPs treatment, however, during the recovery period after flood the expression is reduced but the activity of the enzyme is enhanced [78]. 

The translation initiation is increased due to the upregulation of Poly-A Binding Protein 2 (PAB2) that binds to the poly-A tails of the transcripts and protects it from stress induced degradation and promotes its translation [181]. On the other hand, the transcriptionally regulated genes NmrA-like and FQR1 are members of the NmrA-like family which is an antioxidant ROS scavenging protein family [182]. The NPs alleviate flood stress by regulating protein synthesis, lipid synthesis energy metabolism, and by detoxifying the resulting oxidative stress. Additionally, all these effects were only observed with NPs of size 15 nm. The differentially expressed classes of proteins vary greatly with the size of the NPs [78]. 

#### 6.6.4. Cold Stress

Cold temperatures have a diverse range of physiological damage to plants due to their ability to disrupt protein and lipid structure and integrity. The cold induced lipid modification impacts the membrane integrity and its related functions such as transport and permeability which are essential for plants in photosynthesis, gas exchange and transpiration. Generally, a reduction in transpiration and CO_2_ intake as well as inhibition of chlorophyll is observed. At a molecular level, the large subunit of ribulose-1,5-bisphosphate carboxylase oxygenase (RUBISCO) gets inhibited and consequently its small subunit gets degraded causing a reduction in CO_2_ fixation [183]. When *Cicer arietinum* L. (Chickpea) was treated with TiO_2_NPs under cold stress, it led to differential expression of the genes related to cellular defense, chromatin modification, cell signaling and transcriptional regulation [63]. Most importantly among these were the upregulation of the genes coding for the large and small subunits of RUBISCO, Chlorophyll a/b binding proteins and Phosphoenolpyruvate Carboxylase (PEPC) which in turn led to increased photosynthesis and altered energy metabolism and thus decreased H_2_O_2_ concentration [64]. The higher expression of photosynthetic and energy metabolism proteins alleviates their reduced activity due to cold temperature stress. The energy metabolism is shifted from aerobic to anaerobic pathway and the oxaloacetate formed by the PEPC enzyme is reduced to malate, which is then degraded by dismutation in the mitochondria. Such a metabolic model reduces the electrolyte leakage index [184].

#### 6.6.5. Heat Stress

Numerous genes and transcription factors that are involved in regulating or inducing responses to heat stress are influenced by various NPs [185]. The pretreatment of alfalfa seedling with ZnO NPs before subjecting it to heat stress modified the ultrastructure of chloroplasts, mitochondria, and cell wall thereby averting heat-induced damages and keeping up better plant growth. ZnO NPs considerably improved heat stress-induced reduction of TGS-GUS genes in *Arabidopsis thaliana* seedlings exposed to heat stress (37 °C) [186]. However, the study conducted by Younis et al. [187] found that, *TaPIP1* and *TaNIP2* aquaporin genes were shown to be overexpressed in wheat seedlings when exposed to heat stress when silicon, as opposed to Si NPs, was applied, increasing the relative water content of the plants. 

#### 6.6.6. Heavy Metal Stress

Recent research has shown that plants’ molecular and signaling pathways can be upregulated by using nanoparticles to help them tolerate heavy metals [188]. According to Kareem et al. [189], the upregulation of antioxidant enzymes and osmoprotectants under the influence of ZnO NPs improved heat tolerance mechanisms in mung bean. A dramatic decrease in molecular expression of *IRT1*, *IRT2*, *YSL2*, and *YSL15* genes which are involved in both Fe and Cd absorption, has been observed in rice seedlings after being exposed to ZnVI (100 mg/L). In addition, overexpression of the *OsVIT1* and *OsCAX4* genes resulted in the retention of Cd in vacuoles [190]. Additionally, it was shown that rice plants Si NPs up-regulate the genes for Si uptake (*OsLsi1*) and Cd transport (*OsHMA3*) while down-regulate the genes for Cd uptake and transport (*OsLCT1* and *OsNramp5*) [74]. Venkatachalam et al. [191] found significant genomic modifications in the ZnONPs treated plants, including the appearance of novel DNA bands and/or the absence of typical bands in the RAPD pattern under Cd and Pb stress. Si NPs also suppress the expression of the genes low-affinity cation transporter (LCT1) and natural resistance-associated macrophage protein 5, which are involved in the movement and uptake of Cd from root to shoot (NRAMP5). Both the silicon absorption gene low silicon rice 1 (*LSI1*) and the heavy metal ATPase 3 (*HMA3*) gene, which transports Cd into the vacuoles, are stimulated in rice plants [192]. The expression of antioxidant encoding genes such as *BnGST* (Glutathione S-transferase), *BnPOD* (Peroxidase), and *BnAPX* (Ascorbate peroxidase) that were upregulated under Hg stress were decreased on treatment with sulfur nanoparticles [193].

## 7. The Drawbacks of Using Nanoparticles in Plants

Despite its application in agriculture in combating environmental stress, concerns have been raised about the accumulation of nanomaterials and their potential entry into the food chain [194]. Although it is understood that nanoscale materials such as protein, fat globules, carbohydrates, and DNA present in food are not toxic, the excessive use of certain engineered nanoscale materials in agriculture may pose a threat to both human health and the environment [195]. On exposure to the engineered nanoparticles (ENPs) present in the soil, the plants uptake and bioaccumulate the nanoparticles. Therefore, plants are the fundamental elements of all ecosystems that play a crucial role in the transport of ENPs that bind to the surface of plant roots and can be chemically or physically hazardous to them. Once bound to plant roots, the ENPs may lead to the formation of large pores on cell walls which facilitates the internalization of large ENPs through it [133,196]. It has also been observed in leguminous plants that the nano-cerium oxide particles that entered the roots and root nodules prevented the nitrogen fixing process that soybean crops perform [194]. Concerns are also raised about the chemical damage to the plants and long-term effects of these nanoparticles on the food supply. Further the nanoparticles can gain entry to the food chain, disturb it could be hazardous to humans and animals [197].

## 8. Conclusions and Prospects

Environmental costs brought on by urbanization, harsh weather, habitat loss and global pollution are some of the main factors contributing to abiotic stress, which calls into question the survival of green plants worldwide, including those used in agriculture. In the existing crisis of a population boom, abiotic stressors, such as salinity, high and low temperatures, heavy metals and water scarcity, cast doubts regarding global food security. Nanotechnology is a contemporary technology that has been widely applied in relation to abiotic-stress tolerance in plants. The ability of NPs to shield crop plants from various abiotic challenges and the mechanisms of NP accumulation in plants are both highlighted in this review. Due to the smaller size and high reactivity of nanoparticles, they are easily absorbed by plants in any of their forms, such as nanofertilizers, nanoherbicides or nanopesticides. NPs interact with plants in a variety of ways depending on their chemical makeup, size, surface area and sensitivity. These interactions result in severe morphological, anatomical and physiological changes and are crucial to improving crop plants. Improved growth, biomass production, chlorophyll content, sugar level, accumulation of osmolytes and antioxidant production along with the increased expression of stress-related genes, which elevate protein and chlorophyll content and promote nitrogen metabolism, are some of the beneficial effects of using nanoparticles in abiotic stress management.

Even though the effectiveness of nanoparticles in abiotic-stress tolerance is well documented, most of these studies are still in the laboratory stage. The extensive usage of nanoparticles has prompted worries about their potential adverse effects on the environment and also about the possibility of nanoparticle accumulation in edible plant parts. Hence, focused research aiming to create appropriate evaluation methodologies to evaluate the effects of nanoparticles and nanofertilizers on biotic and abiotic ecosystem components is needed. In addition, the impact of nanoparticles on human beings and identification of the acceptable limits are also necessary. Future studies should concentrate on designing NPs that are affordable, nontoxic, ecologically safe and self-degradable in order to commercialize nanotechnology from the laboratory to the agricultural fields.

## Figures and Tables

**Table 1:** Morphological changes under the influence of nanoparticles.

Stress	Plant	Nanoparticles	Morphological Changes under the Influence of Nanoparticles	Reference
Drought	*Brassica napus* L.	Fe	Increased biomass production and leaf growth	[91]
Salt	*Dracocephalum moldavica* L.	Ti	Increased plant height	[90]
Flood	*Glycine max* (L.) Merr	Al_2_O_3_	Enhanced seedling weight and root length	[77]
Salt	*Sorghum bicolor* (L.) Moench	Zn	Improved shoot length and root length	[92]
Heat	*Triticum aestivum* L.	Ag	Improved plant growth, root and shoot length, dry weight and fresh weight	[84]
High temperature	*Sorghum bicolor* (L.) Moench	Se	Increased pollen germination	[83]
Drought	*Triticum aestivum* L.	Se	Improved shoot and root length, leaf area and leaf number	[93]
Salt	*Moringa oleifera* Lam.	Fe_3_O_4_	Improved plant growth, number of branches, leaf area and biomass	[93]
Drought	*Oryza sativa* L.	ZnO	Increased plant height, fresh weight and dry weight	[94]
Drought	*Linum usitatissimum* L.	Fe_2_O_3_	Enhanced growth parameters, such as shoot and root length and seed yield	[95]
Salt	*Lycopersicon esculentum* Mill.	Si	Retained fruit quality and size	[96]
Cadmium and drought stress	*Triticum aestivum* L.	Fe	Improved photosynthesis and yield	[97]

**Table 2:** Physiological changes under the influence of nanoparticles.

Stress	Plant	Nanoparticle	Physiological Response of Plants under the Influence of Nanoparticles	Reference
Heavy metal (lead)	*Coriandrum sativum* L.	Si	Reduced MDA	[110]
Drought	*Linum usitatissimum* L.	Ti	Reduced chlorophyll damage, electrolyte leakage, lipid peroxidation and H_2_O_2_ accumulation	[111]
Drought	*Brassica napus* L.	Fe	Reduced MDA production	[91]
Salt	*Dracocephalumm oldavica* L.	Ti	Enhanced nutrient uptake	[90]
Salt	*Medicago sativa* L.	K_2_SO_4_	Reduced electrolyte leakage	[49]
Salt	*Moringa oleifera* Lam.	Fe_3_O_4_	Improved photosynthesis and decreased lipid peroxidation	[93]
Salt	*Triticum aestivum* L.	Au	Improved nitrogen metabolism	[112]
Salt	*Abelmoschus esculentus* (L.) Moench	Zn	Enhanced photosynthetic pigments	[113]
Drought	*Linum usitatissimum* L.	Fe_2_O_3_	Reduced MDA and H_2_O_2_ accumulation	[95]
Drought	*Oryza sativa* L.	ZnO	Decreased lipid peroxidation	[94]
As stress	*Hordeum vulgare* L.	CaO	Enhanced Ca uptake, reduced As uptake and accumulation	[114]

**Table 3:** Biochemical changes under the influence of nanoparticles.

Stress	Plant	Nanoparticles	Biochemical Changes	Reference
Drought	*Linum usitatissimum* L.	Ti	Improved protein and seed oil production	[111]
Drought	*Oryza sativa* L.	ZnO	Enhanced proline content	[94]
Salt	*Pennisetum gluacum* (L.) R.Br.	Ag	Proline, ROS and MDA reduced	[50]
Salt	*Medicago sativa* L.	K_2_SO_4_	Increased proline content	[49]
Salt	*Lycopersicon esculentum* Mill.	Si	Improved chlorophyll and phenol contents	[96]
Salt	*Moringa oleifera* Lam.	Fe_3_O_4_	Increased crude protein, fiber and minerals	[93]
As stress	*Oryza sativa* L.	Fe	Enhanced accumulation of proline, glutathione and phytochelation	[12]
Salt	*Abelmoschus esculentus* (L.) Moench	Zn	Reduced proline content	[113]

## Data Availability

Not applicable.
